# Brief disruptions in capillary flow result in rapid onset of hypoxia

**DOI:** 10.1117/1.NPh.12.S2.S22803

**Published:** 2025-08-12

**Authors:** John T. Giblin, Sreekanth Kura, Gülce Küreli, John Jiang, Kıvılcım Kılıç, Baoqiang Li, Sava Sakadžić, Anna Devor, David A. Boas

**Affiliations:** a Boston University, Department of Biomedical Engineering, Boston, Massachusetts, United States; b Boston University, Neurophotonics Center, Boston, Massachusetts, United States; c Massachusetts General Hospital, Harvard Medical School, Athinoula A. Martinos Center for Biomedical Imaging, Department of Radiology, Charlestown, Massachusetts, United States; d Brain Cognition and Brain Disease Institute, Shenzhen Institutes of Advanced Technology, Chinese Academy of Sciences; Shenzhen-Hong Kong Institute of Brain Science, Shenzhen Fundamental Research Institutions, Shenzhen, Guangdong, China

**Keywords:** capillary stalling, cerebral oxygen, phosphorence lifetime, two photon microscopy

## Abstract

**Significance:**

Capillaries are the critical site of vascular exchange with the local tissue, with continuous flow to meet the brain’s unique and steep energetic demands. However, transient stalls in capillary flow have been observed and at elevated levels in preclinical models of disease. Systematic measurements have not been made to quantify the acute effects of individual capillary stalls on local oxygen.

**Aim:**

We aim to quantify oxygen dynamics around capillary stalls as they occur *in vivo.*

**Approach:**

We use high-resolution two-photon phosphorescent lifetime microscopy (2PLM) to monitor capillary flux and ${\mathrm{pO}}_{2}$ in the mouse cortex, allowing us to capture acute oxygen dynamics around capillary stalling.

**Results:**

All stalls cause rapid drops in intra-capillary oxygen that likely extend to local tissue based on estimates using the erythrocyte-associated transient (EAT). This includes a subset of capillaries, which reach critically hypoxic levels (<10 mmHg), which could not be predicted by the capillaries’ normal flux and oxygen levels, nor local vessel density or proximity to diving arterioles and venules.

**Conclusion:**

Our findings indicate that a subset of capillary stalls reach extremely low local oxygen, resulting in transient hypoxia in the surrounding tissue. This reveals a new potential pathological mechanism due to stalled capillary flow.

## 1 Introduction

Cerebral blood flow relies on continuous perfusion of capillaries to distribute and deliver oxygen and other nutrients, with very little energy reserves to tolerate disruptions in flow.^1^^,^^2^ Recently, such disruptions have not only been observed at the capillary level, but observed with increased frequency in models of Alzheimer’s disease^3^ and stroke,^4^^,^^5^ and mild traumatic brain injury (mTBI).^6^ Both modeling and measurement have shown that these events are disruptive to cerebral blood flow at the network scale.^7^^–^^9^ However, the acute effects of a single stall in capillary flow have not been well investigated. As blood flow is stopped for the duration of the stall, there is potential for acute hypoxia, although this has not been directly measured aside from anecdotally.^5^^,^^10^ However, a recent study using a bioluminescent oxygen indicator associated hypoxic pockets with the nearby presence of injected microspheres,^11^ offering strong evidence of stall-associated hypoxia. As stalls can have durations that are minutes long,^3^^,^^5^^,^^12^ and occur repeatedly in the same capillaries, there is the potential for extended and repeated exposure to low levels of oxygen. Further, heterogeneous flow and vascular density in the microvascular network result in cortical tissue oxygen also being highly variable,^13^ meaning stalls could have differing impacts depending on their location in the network. Together, this raises the question of to what degree individual stalls acutely affect oxygen delivery in the brain and if this could be a part of their contribution to pathology.

Two-photon phosphorescent lifetime microscopy (2PLM) offers high-resolution measurement of the partial pressure of oxygen (${\mathrm{pO}}_{2}$)^14^^,^^15^ deep into the brain, enabling investigations into the distribution and consumption of oxygen.^10^^,^^16^ In addition to precise measurements of oxygen, the lack of uptake of the dye by circulating red blood cells means that capillary flux^10^^,^^17^ and speed can be estimated from capillary point measurements. The inter-RBC ${\mathrm{pO}}_{2}$ measured via erythrocyte-associated transient (EAT) has also been shown to serve as a useful proxy for local tissue ${\mathrm{pO}}_{2}$.^18^ These characteristics make 2PLM an ideal tool for studying stalling, with both the spatial and temporal resolution (when using single point measurements) to capture stalls.

To quantify the effect of stalling on oxygen, we monitor capillary oxygen, flux, and speed in 10 to 20 capillaries for ∼10 min to capture instances of capillary stalling. We repeat these 10-min recordings in several different regions of interest in several different mice.

We use the inter-RBC ${\mathrm{pO}}_{2}$ during normal flow to demonstrate that the stalling-induced drop in oxygen likely extends out to the local tissue, implying that capillary stalls create local tissue hypoxia in some cases. We also tried to predict oxygen levels during a stall based on capillary oxygen during normal flow, capillary flux during normal flow, or local vascular density and found they did not explain the difference in oxygen levels during a stall. As stalls often occur frequently in the same capillaries,^19^ this demonstrates the potential for repeated hypoxic stress due to capillary stalling.

## 2 Materials and Methods

### 2.1 Animal Preparation

All animal procedures were approved by the Boston University Institutional Animal Care and Use Committee and were conducted following the Guide for the Care and Use of Laboratory Animals. Animals underwent surgery for headpost implantation and craniotomy as described previously.^20^^,^^21^ Animals were allowed to recover for 2 weeks and then underwent acclimation to head fixation. To acclimate the animal to awake head fixation, they were briefly anesthetized with 3% isoflurane and placed in a custom cradle. Sessions started at 15 min and were repeated once daily with increasing durations until animals were comfortable with a 90-min session. Sweetened condensed milk was given by a transfer pipette as a reward. If animals exhibited distress at any time in any session, they were removed from the cradle.

### 2.2 Two-Photon Microscopy

A Bruker investigator was used with an integrated photon counting card (Becker and Hickl 150N) for lifetime measurements. Phosphorescence was excited at 950 nm with a pulse laser (Insight X3, Spectra Physics, 80 MHz repetition rate ∼120  fs pulse width), with excitation power controlled with an electro-optic modulator (EOM) (Conoptics 350-105). The beam was focused and emission collected through a water immersion objective (Nikon 16× 0.8 NA). The excitation and emission light were split with a primary dichroic (900 SP) and excess laser power blocked by an 890 SP filter in the detection path. Photons were detected by a photomultiplier tube (Hamamatsu H10770PA-50) after passing through a secondary dichroic (565 LP) and emission filter (795/150).

### 2.3 Phosphorescent Lifetime Imaging

Mice were first briefly anesthetized with 3% isoflurane and injected with 0.1 mL Oxyphor-2P iv (5  mg/mL in PBS) before being head fixed and allowed to recover. Once recovered, the animals were placed under the microscope, and the water between the objective and cranial window was heated to 35°C to 37°C by an objective heater (Bioscience Tools TC-HLS-05).^22^^,^^23^ An 811×811  μm region of interest was selected for imaging, and in some cases, this was restricted to 405×405  μm. Imaging planes started at ∼50  μm below the cortical surface and were separated by 50  μm down to an approximate depth of 250 to 300  μm. In each plane, 10 to 20 points were selected based on capillaries with clear cross sections parallel to the imaging plane. Capillaries were identified based on their diameter and morphology. At each point, 1000 cycles of 10  μs excitation and 290  μs of collection and photon counting were performed for a total of 300  ms/point. The points were iterated through repeatedly for 9 to 10 min. The raw photon counting data was saved for offline analysis. We measured ∼800 capillaries across seven awake mice, which yielded 216 capillaries with sufficient signal to noise to track both red blood cell flux and ${\mathrm{pO}}_{2}$. We also measured an additional three mice under anesthesia for comparison, which yielded 114 capillaries. See Table 1 in Appendix for a complete summary.

### 2.4 Measurement of Capillary ${\mathrm{pO}}_{2}$, Flux, and EAT

For the calculation of intracapillary oxygen, raw photon counting files were used to generate sdt files with Bruker’s Image Ripping Utility. The processed sdt files contain the averaged decay curve generated from the 1000 measurement cycles every time a point was visited. The ${\mathrm{pO}}_{2}$ was calculated in a similar manner described previously.^10^^,^^14^ Briefly, the phosphorescent decay was fitted to a single exponential decay to generate a lifetime estimate, and the first 5  μs of the 290  μs decay was discarded to account for the electro-optic modulator (EOM) response time. The lifetime was then converted to ${\mathrm{pO}}_{2}$ based on a Stern-Volmer type relation, calibrated by an oxygen titration experiment for every batch of dye.

Phosphorescent intensity during the entire 300  μs measurement cycle was generated by parsing and converting the raw photon count data using custom MATLAB code. This generated a 300-ms intensity-time course for each point each time it was visited. Intensity traces were manually inspected for clear dips in intensity that corresponded to the passage of RBCs. Selected capillaries and time points were then binarized to determine the period of plasma or RBC passage using a similar approach as others^10^^,^^17^^,^^18^^,^^22^ based on Otsu’s method.^24^ Measurements and time points with a substantial drop in photon counts per cycle or loss of clear flux signal-to-noise ratio were rejected. The binarized trace was then used to count the number of RBCs present to calculate flux.

For the EAT profile calculation [Fig. 3(a)], the RBC locations in the 300-ms time course were used to re-bin all the photon arrival times based on proximity to each RBC. Distance was calculated as the time to the nearest RBC passage times its speed.

### 2.5 Calculation of Depth and Vessel Density

To estimate both local vessel density and the stall’s proximity to nearby arterioles and venules, measurement points were first manually identified by comparing planar reference images taken just before PLIM measurements to high-quality fluorescent angiograms taken on a separate day. The coordinates, including depth from the surface, were saved and used to generate subvolumes cropped 75 pixels in all directions around the measurement point. Subvolumes were smoothed using a 3×3×3 median filter and binarized before being skeletonized to obtain vessel centerlines. The Euclidean distance from the PLIM point measurement to every pixel classified as part of a vessel in the reference angiogram was then calculated and binned into a histogram with 1-μm bin widths. This histogram was used as an estimate for local vessel density (reported in arbitrary units) as a function of distance from the PLIM point where a given stall occurred, where the vessel density is represented by the number of pixels binarized as vessels. For nearest arteriole and venule distances, measurements were manually taken between the measured PLIM point and the edge of the closest diving or pial vessel. Arterioles and venules were differentiated by morphology in the angiograms, with a bright field image of the cranial window providing additional guidance as needed.

## 3 Results

### 3.1 Capillary Stalls Cause a Rapid Drop in Oxygen

**Fig. 1:**
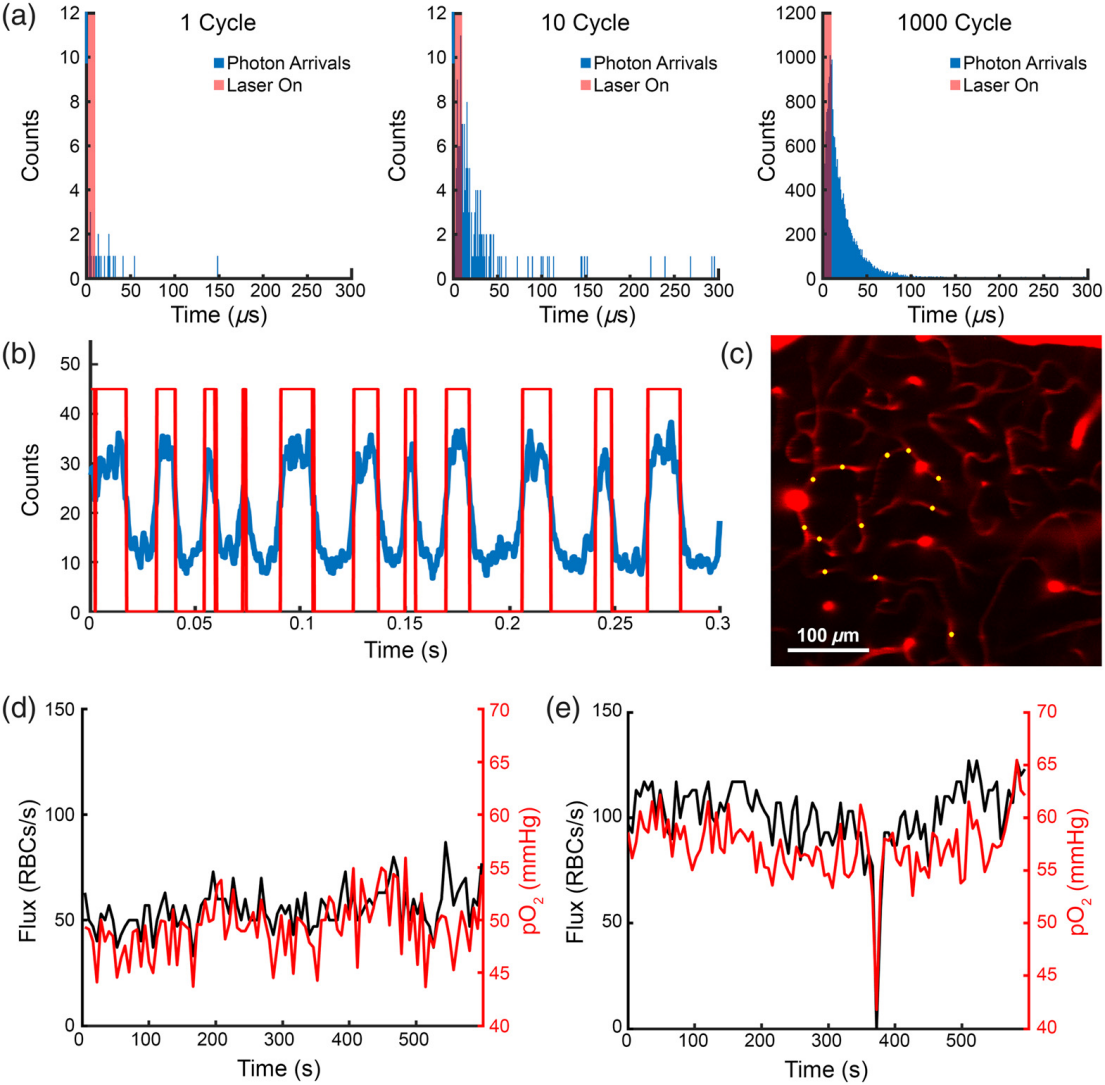
(a) Buildup of phosphorescent lifetime decay over repeated 300  μs cycles of 10  μs excitation and 290  μs of detection. (b) The same cycles plotted as intensity (counts/cycle) versus time, showing dips during times of RBC passage. (c) Placement of PLIM measurement points on capillaries in a reference scan. Scale bar: 100  μm. (d) Representative flux and ${\mathrm{pO}}_{2}$ trace of a flowing capillary. (d) Representative flux and ${\mathrm{pO}}_{2}$ trace of a stalling capillary showing that ${\mathrm{pO}}_{2}$ drops when flux goes to 0.

We used the recently developed, two-photon optimized, phosphorescent probe Oxyphor 2-P to monitor capillary flux and oxygen in sets of capillaries for ∼10  min at a time and capture stalling events.^25^ The same point measurements could be used to build both lifetime decays [Fig. 1(b)] and intensity time courses [Fig. 1(c)], where dips in intensity correspond to red blood cells, which do not take up the phosphorescent probe. This allowed for a robust oxygen and flux estimate to be obtained within 300 ms at each capillary, and a set of capillaries to be monitored at a rate of roughly 0.1 to 0.2 Hz, sufficient to observe spontaneous stalls in flow [Fig. 1(d)]. We performed these measurements down to 300  μm in depth in middle-aged mice (n=7 animals, aged 7 to 10 months), capturing instances of stalled flow [Fig. 1(e)] and the associated changes in capillary oxygen.

**Fig. 2:**
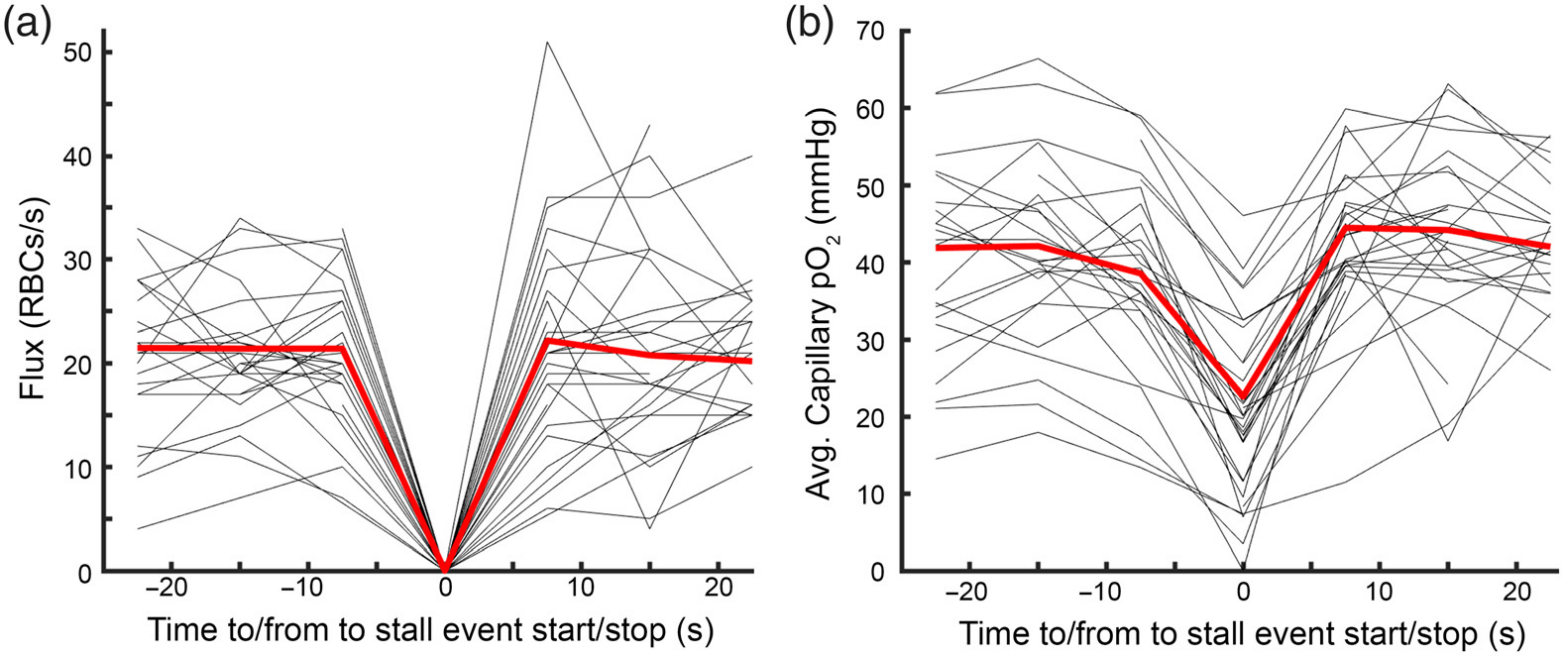
(a) RBC flux around stalling events, t=0 indicates the time the capillary stalled, where fluxis 0 red blood cells (RBCs/s). (b) Oxygen dynamics around stalling events, showing a characteristic drop in oxygen as a result of the stall (t=0), which then recovers as soon as flow resumes (t>0). Most stalling events lasted for a single time point (Fig. 8 in Appendix), but for the stalls that spanned multiple time points, oxygen levels were averaged over the duration of the stall event to a single value represented at t=0.

As flux and oxygen are tightly correlated, we consistently observed a distinct drop in intra-capillary oxygen for the duration of the stall [Fig. 2(b)], followed by rapid recovery back to normal levels on the resumption of flow. Most of the stalls we captured lasted for just a single time point [Fig. 8(c) in Appendix], and the few capillaries that stalled for a longer duration tended to stay at the lower oxygen level [Figs. 8(a)–8(b) in Appendix]. Although our temporal resolution was not sufficient to be certain that stalls were continuous and that there were not brief periods of flow between measurement time points. We also did not see a significant difference in flux and oxygen before and after the stall [Figs. 2(a)–2(b)]. We found that this drop in oxygen was consistent across every stall we measured [Fig. 2(b)] and that 40% (13/32) of stalls observed resulted in intracapillary oxygen dropping to hypoxic levels below 10 mmHg,^26^ with 25% (8/32) falling below 5 mmHg. The mean ${\mathrm{pO}}_{2}$ during a stall overall was 15.4±11.6  mmHg.

### 3.2 Capillary Stalling Affects Local Tissue Oxygen

**Fig. 3:**
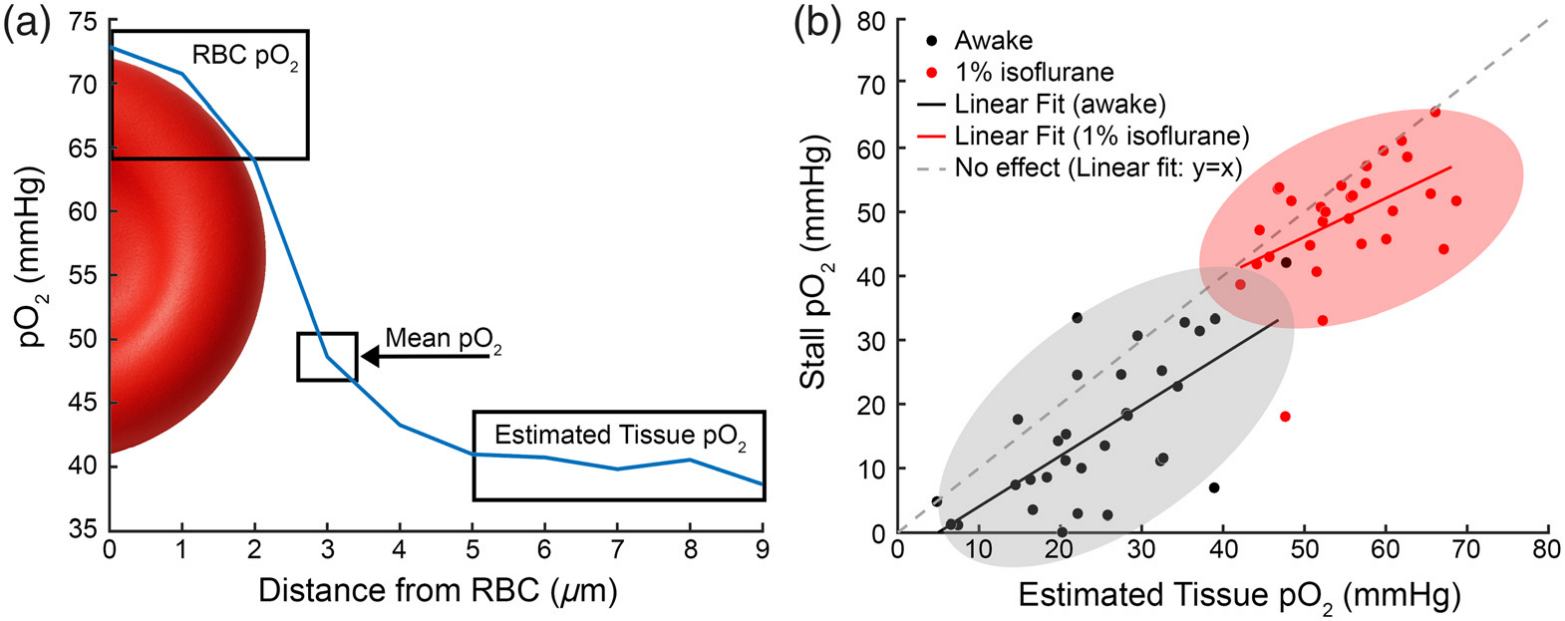
(a) Schematics of average oxygen profile from a representative capillary, calculated based on the time from the nearest RBC passage and its speed. ${\mathrm{pO}}_{2}$ far away from the RBC is used as an estimate of the tissue ${\mathrm{pO}}_{2}$ surrounding the capillary. (b) Comparison of stall ${\mathrm{pO}}_{2}$ to the estimated tissue ${\mathrm{pO}}_{2}$ calculated when the capillary was flowing, showing that most stalls affect local tissue ${\mathrm{pO}}_{2}$. There is a tendency for this drop to be less severe under anesthesia (red dots) compared to awake (black dots). Anesthesia fit: y=0.61x+16, $R^{2}=0.24$. Awake fit: y=0.79x−4, $R^{2}=0.47$.

To better understand how this drop in oxygen dynamics could affect the surrounding tissue, we used the EAT to estimate the local tissue oxygen during normal flow [Fig. 3(a)].^18^ Using the inter-RBC ${\mathrm{pO}}_{2}$ as an estimate for local oxygen (see Methods for details), we compared estimated tissue ${\mathrm{pO}}_{2}$ during fluxing ${\mathrm{pO}}_{2}$ with levels during the stall, where intra-capillary ${\mathrm{pO}}_{2}$ is likely at equilibrium with local tissue due to a lack of RBC passage. We saw that the intracapillary oxygen during the stall typically fell below the inter-RBC ${\mathrm{pO}}_{2}$ as calculated using the EAT [Fig. 3(b)], implying that oxygen in the capillary during the stall was lower than the surrounding tissue oxygen during normal flow. This demonstrates that not only does capillary oxygen drop during loss of flow as expected, but this drop also appears to extend to the immediate surrounding tissue that is supplied by the capillary. Further, this drop was observed to reach a lower steady state in long stalling capillaries, so this lower oxygen level can be assumed to be maintained for the duration of the stalling event. We also took similar measurements in a set of similarly aged animals (n=3) under 1% isoflurane anesthesia. We see that no stalls appear to generate hypoxia, likely due to vasodilation and reduced consumption due to isoflurane.

### 3.3 Factors Determining Stall Hypoxia

**Fig. 4:**
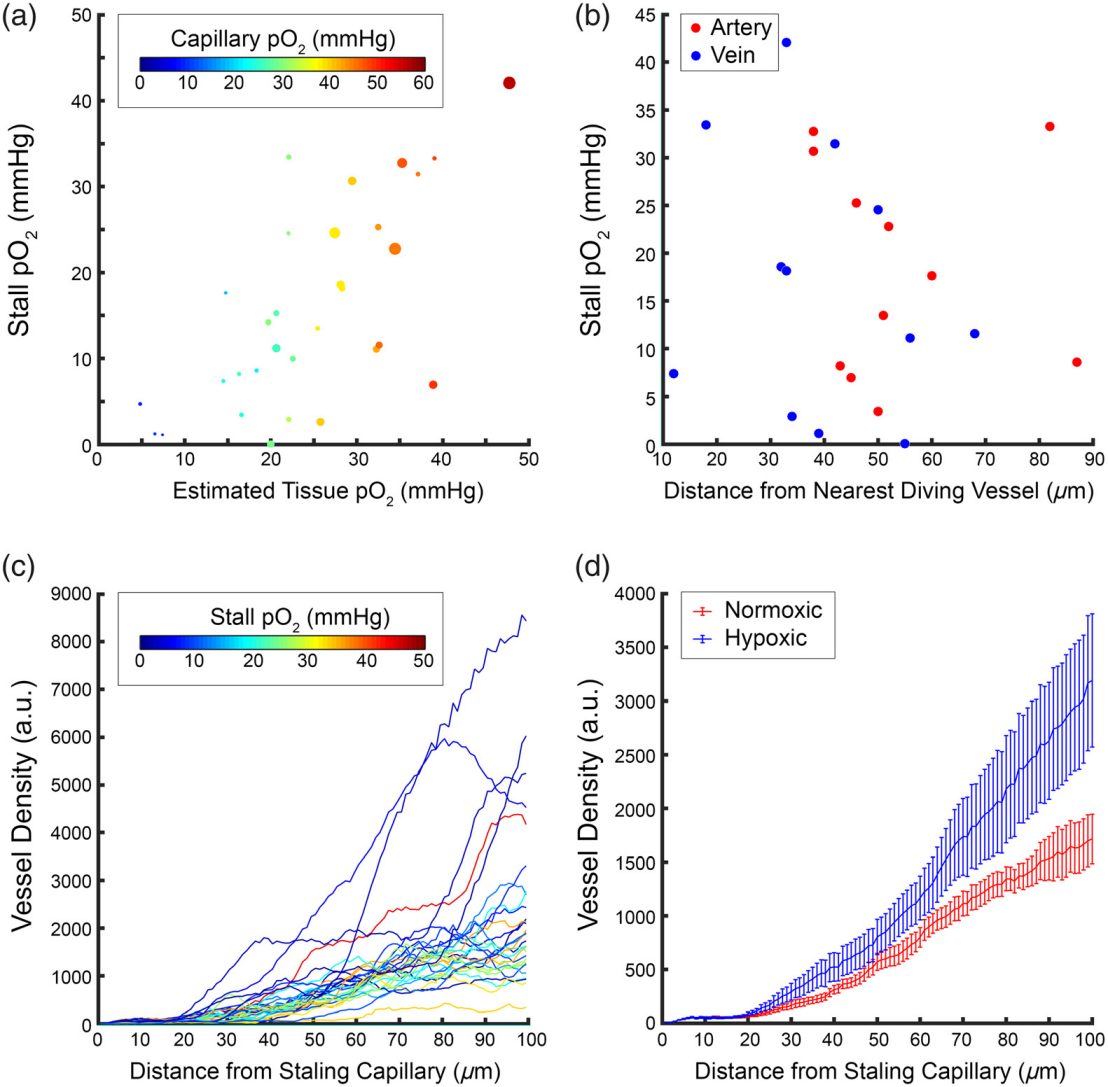
(a) Comparison of stall ${\mathrm{pO}}_{2}$ with estimated tissue ${\mathrm{pO}}_{2}$, capillary ${\mathrm{pO}}_{2}$, and flux during normal flow. Colors indicate capillary ${\mathrm{pO}}_{2}$ during non-stall periods, and the diameter of each point is proportional to RBC flux. (b) ${\mathrm{pO}}_{2}$ during a stall versus the manually measured distance to the nearest diving artery or vein. (c) Estimated vessel density versus distance from stall. Vessel density is estimated from the number of pixels in a binarized reference angiogram. Each line represents a different stalling vessel; color indicates the mean ${\mathrm{pO}}_{2}$ level during the stalling event. (d) Comparison of average density counts between stalls that resulted in ${\mathrm{pO}}_{2}$ above (normoxic-red) or below (hypoxic-blue) 10 mmHg.

We saw that capillaries that had similar tissue ${\mathrm{pO}}_{2}$ [Fig. 3(b)] during normal flow could have a dramatically different ${\mathrm{pO}}_{2}$ during stalling. To better understand this relationship, we examined whether flux or capillary ${\mathrm{pO}}_{2}$ determined whether a stall resulted in hypoxia. However, we observed that there was a mix of high and low flux capillaries that were hypoxic during stalling [Fig. 4(a)] and that the drop in ${\mathrm{pO}}_{2}$ varied across capillary ${\mathrm{pO}}_{2}$ and estimated tissue ${\mathrm{pO}}_{2}$. To further investigate, we measured the distance between the stalling capillary and the nearest diving vessel [Fig. 4(b)], which could serve as a large source of oxygen to avoid hypoxia during stalls. There was no clear trend between stall hypoxia and proximity to arteries and veins. We also measured vessel density from a binarized volume taken in a 225×225×150  μm volume centered around the measurement point (see Sec. 2). This was then used to compare vessel density as a function of distance from the stalling capillary in both hypoxic (≤10  mmHg) and normoxic stalls. Looking at all density estimates, the hypoxic stalls tended to have similar density counts close to the point but diverged further away [Fig. 4(d)]. Although this seemed to be primarily driven by a subset of hypoxic stalls that were close to pial veins [Fig. 4(c)]. We also did not see any relationship between stalling and cortical depth (Fig. 6 in Appendix), so the divergence in estimated vessel density was not driven simply by the cortical depth of the stall.

### 3.4 Effects of Stalling on Nearby Capillary Oxygen

**Fig. 5:**
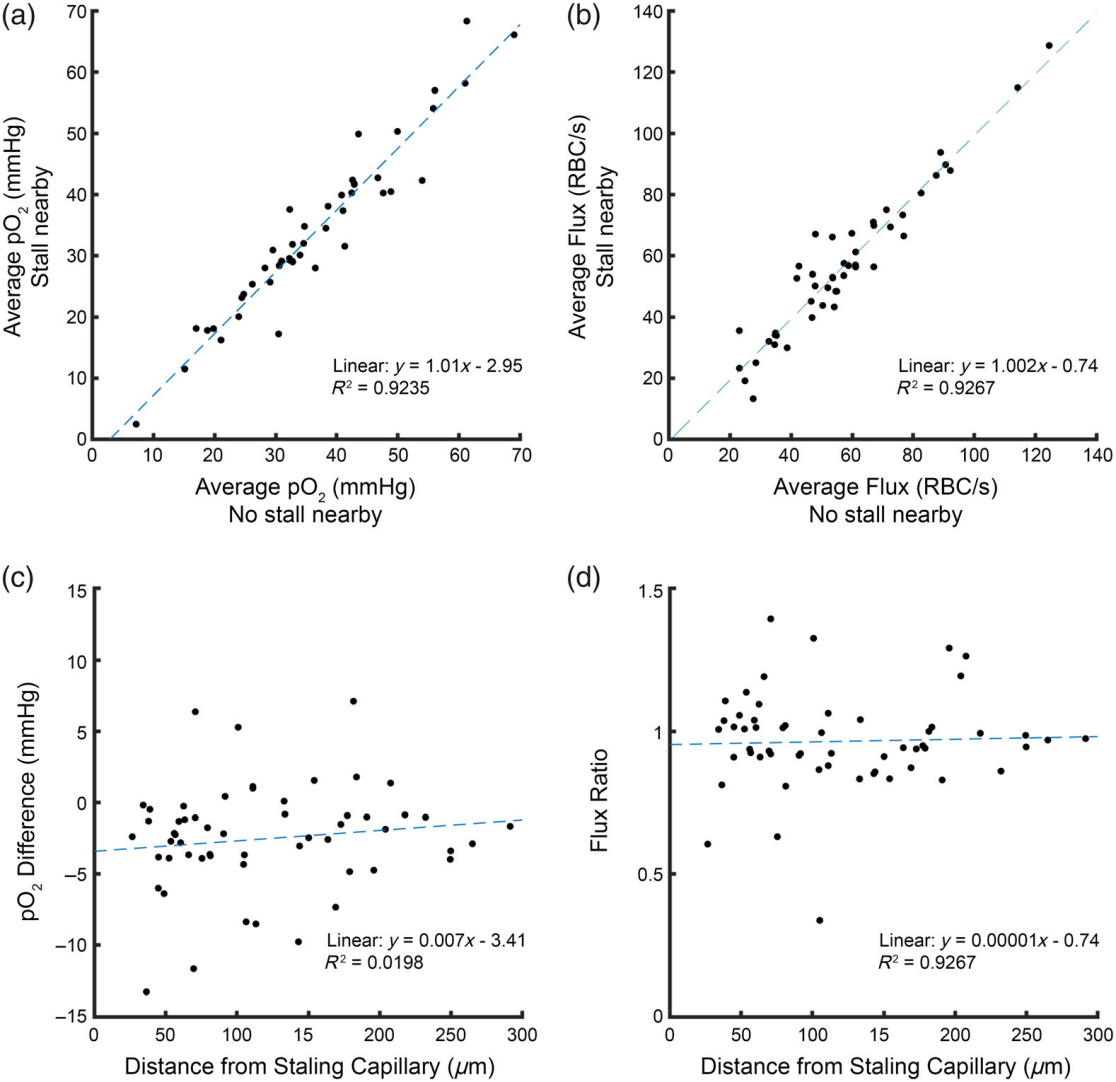
(a) Comparison of capillary oxygen when a stall was occurring nearby versus when no stall was detected. (b) Comparison of capillary flux when a stall was occurring nearby versus when no stall was detected. (c) Change in capillary oxygen due to nearby stalling as a function of distance from the stalling capillary. (d) Change in capillary flux due to nearby stalling (stall/no stall) as a function of distance from the stalling capillary. Dashed lines in all plots show the linear fit.

After establishing the drop in oxygen in the immediate stall vicinity, we next looked at capillaries that maintained flow throughout the measurements but were measured alongside a capillary that underwent stalling. Looking across all capillaries, there was no consistent change in oxygen [Fig. 5(a)] or flux [Fig. 5(b)] as a result of being in proximity to a stall. Although there did appear to be a subset of capillaries that had either an increase or decrease in flux or oxygen when compared with when there was no stall detected. The decrease in partial oxygen pressure seemed to be largest close to the capillary that stalled [Fig. 5(c)], which would be consistent with the idea that there is a transient sink of oxygen around a stall, pulling oxygen from nearby tissue or capillaries to compensate. On average, there was a modest decrease in oxygen (−2.5  mmHg), and flux (96%) [Fig. 5(d)] compared with when no stall was detected, although it is always possible that there were other stalls nearby that were not detected, which could confound these estimates.

## 4 Discussion

We measured capillary oxygen and RBC flux in sets of capillaries for long time periods to capture instances of stalled flow. We found that stalling unsurprisingly always results in a drop in the intracapillary oxygen pressure, usually to lower levels than the inter-RBC ${\mathrm{pO}}_{2}$ during normal flow estimated using the EAT. This indicates that even brief stalling events (in the order of a few seconds) can still result in a substantial drop in intracapillary oxygen levels, which also likely extends to local tissue oxygen levels. More surprisingly, we found that even in healthy mice, a substantial portion of stalled (13 out of 32 stalls) capillaries drop to hypoxic levels because of the stall [Fig. 2(b)]. We selected 10 mmHg as our hypoxic threshold because below this cell respiration rate is dependent on oxygen concentration,^26^ although the breakdown in mitochondrial function may be lower.^27^ However, we still saw a number of capillaries (8/32) fall below 5 mmHg as well. This hypoxia was not seen in animals under isoflurane anesthesia, due to higher flow and presumably lower consumption,^22^ highlighting that continuous capillary flow is often critical to keep up with resting metabolism in awake animals. Our findings are consistent with a recent study^11^ that showed the presence of transient, spatially constrained hypoxic pockets using a bioluminescent oxygen reporter. These hypoxic events were confined to ∼45  μm, lasted anywhere from seconds to minutes, and were often associated with the presence of injected fluorescent microspheres, which were presumably obstructing a capillary nearby. Our current work offers a mechanistic explanation for these events, directly linking them to spontaneous stalling of individual capillaries and showing that such stalls can drive acute intracapillary and local tissue hypoxia. We also observe similar mitigation of hypoxia under isoflurane anesthesia and found that neither estimated vascular density nor capillary proximity to larger vessels predicts hypoxic severity, in agreement with the spatial unpredictability reported by Beinlich et al.^11^ Their results also show that a mobile animal on a running ball had even fewer hypoxic pockets, which could help explain our observed variability in oxygen during stalls, although it would need to be confirmed with behavioral data which our study lacks.

Flux and capillary ${\mathrm{pO}}_{2}$ during normal flow did not appear to be predictive of the drop in ${\mathrm{pO}}_{2}$ as a result of stalling, or whether or not the stall ${\mathrm{pO}}_{2}$ became hypoxic. Hypoxia was also not predicted by the depth of the stalled capillaries below the cortical surface. Finally, when looking at estimates of local vessel density, we did not find lower density in the proximity of more hypoxic stalls. These structural metrics may not be sufficient to explain the variation in the local vascular network properties, for example, we were not able to measure the branch order of capillaries from arteries and veins or the branch distance between capillaries. In modeling of cerebral capillary flow, local vascular topology has been shown to be critical in the magnitude of the impact,^28^ although we measured a more substantial drop in oxygen than predicted by modeling.

We also compared flux and ${\mathrm{pO}}_{2}$ in capillaries that did not stall but were measured at the same time as a capillary that did stall. On average, flux and oxygen were reduced when a nearby capillary was stalled compared with when it was flowing, but the average change was small (2.5 mmHg and 4% reduction in RBC flux). However, there were a few local capillaries that saw drops as large as 13 mmHg during the stall period. In addition, we also saw that a few capillaries had a flux or ${\mathrm{pO}}_{2}$ increase during the stall. This could potentially be because the capillary was part of a downstream flow path that saw increased or decreased flux as a part of redistributed flow around the stall.^28^ We also observed anecdotally that complete stalling of capillary flow was not necessary to create transient hypoxia (Fig. 7 in Appendix). In this example, there was no stall detected during the periods of reduced flow (though it is possible it occurred outside our measured capillaries). A significant drop in flux, even if flux is not fully stopped, was still sufficient to create critically low oxygen in this case. Further, due to the phase separation effect^29^^–^^31^ in the blood, downstream occlusions do have the potential to disrupt hematocrit as well. This is also in line with the modeling of single capillary occlusions, which showed both flow and RBC flux are significantly reduced up to 3 to 4 branches up and downstream.^28^ Therefore, in addition to local hypoxia generated by the stalling event itself, its detrimental effect on local flow patterns could also be sufficient in some circumstances to create pockets of hypoxia around nearby capillaries despite their continuous flow. According to modeling of stalls, the magnitude and range of this impact are determined by the local vascular topology.^28^ This will likely be in capillaries far from diving vessels along long path lengths, which would be the most vulnerable to hypoxia.^32^^,^^33^

In summary, we demonstrated that stalls in capillary flow predictably result in a drop in oxygen, showing evidence that individual stalling events are potentially detrimental to local tissue. Because the same subset of capillary stalls repeatedly, this also means that these drops would be repeatedly occurring over long periods of time. Further, with the increased incidence of stalling that occurs with age or disease such as Alzheimer’s^3^ or stroke,^4^^,^^5^^,^^12^ there is increased vulnerability to acute metabolic disruption. Future work could extend these measurements to deeper layers, as well as comparisons to disease models. Flow and oxygen distributions are different across brain^10^^,^^22^^,^^34^ regions and could potentially result in different dynamics around stalling events. These variations could impact how often a stall results in hypoxia. Furthermore, we used inter-RBC ${\mathrm{pO}}_{2}$ as an indicator of local tissue ${\mathrm{pO}}_{2}$. Although previous work has shown that they have been shown to be comparable, their accuracy may vary across brain regions or local vascular topology. Future experiments could be designed to directly measure tissue oxygen with Oxyphor-2P injected into the CSF, with a vascular tracer used to monitor nearby capillary stalls.^16^ Finally, investigating how the mouse’s behavioral state relates to hypoxic stalls would also be critical in understanding our observed variability in oxygen levels.

## 5 Appendix: Supplemental Information

**Fig. 6:**
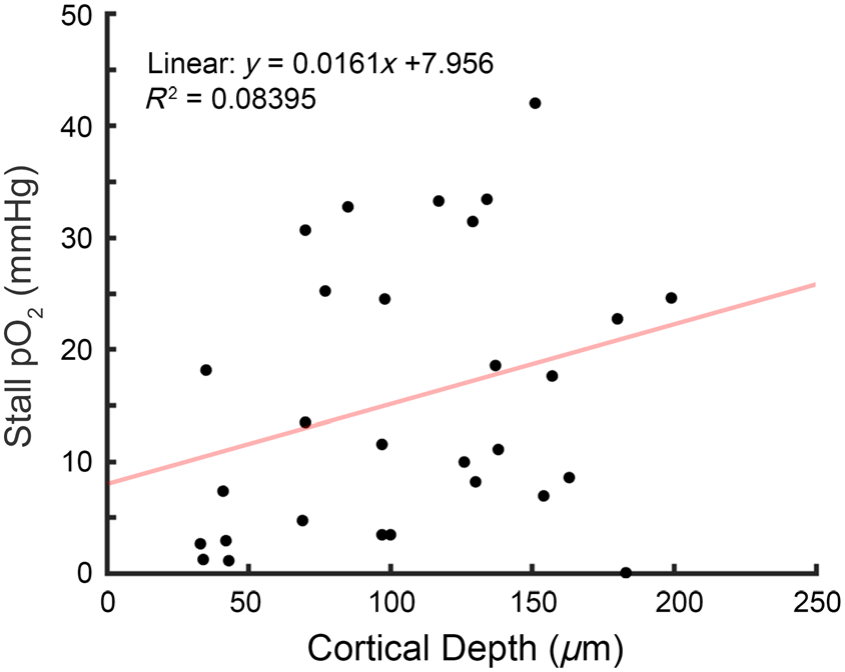
Comparison of stall ${\mathrm{pO}}_{2}$ based on estimated depth below pial surface, showing it is not predictive of stall hypoxia. Depth was determined by manually finding points in a high-quality reference angiogram.

**Fig. 7:**
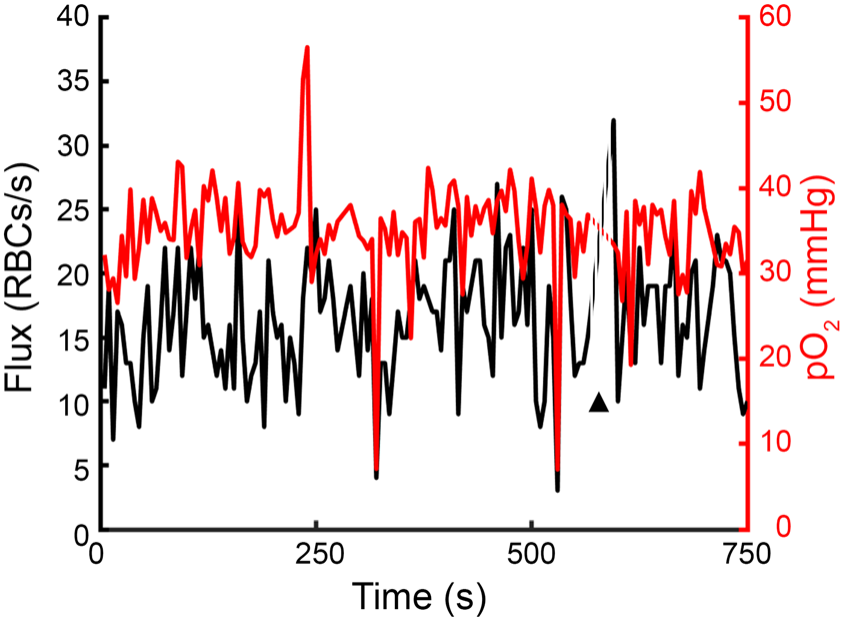
Time trace showing flux and ${\mathrm{pO}}_{2}$ in a capillary across time, showing ${\mathrm{pO}}_{2}$ drops well below 10 mmHg when flux is low but not completely stalled. Black arrow and dashed lines represent a break in the measurements due to loss of adequate signal quality.

**Fig. 8:**
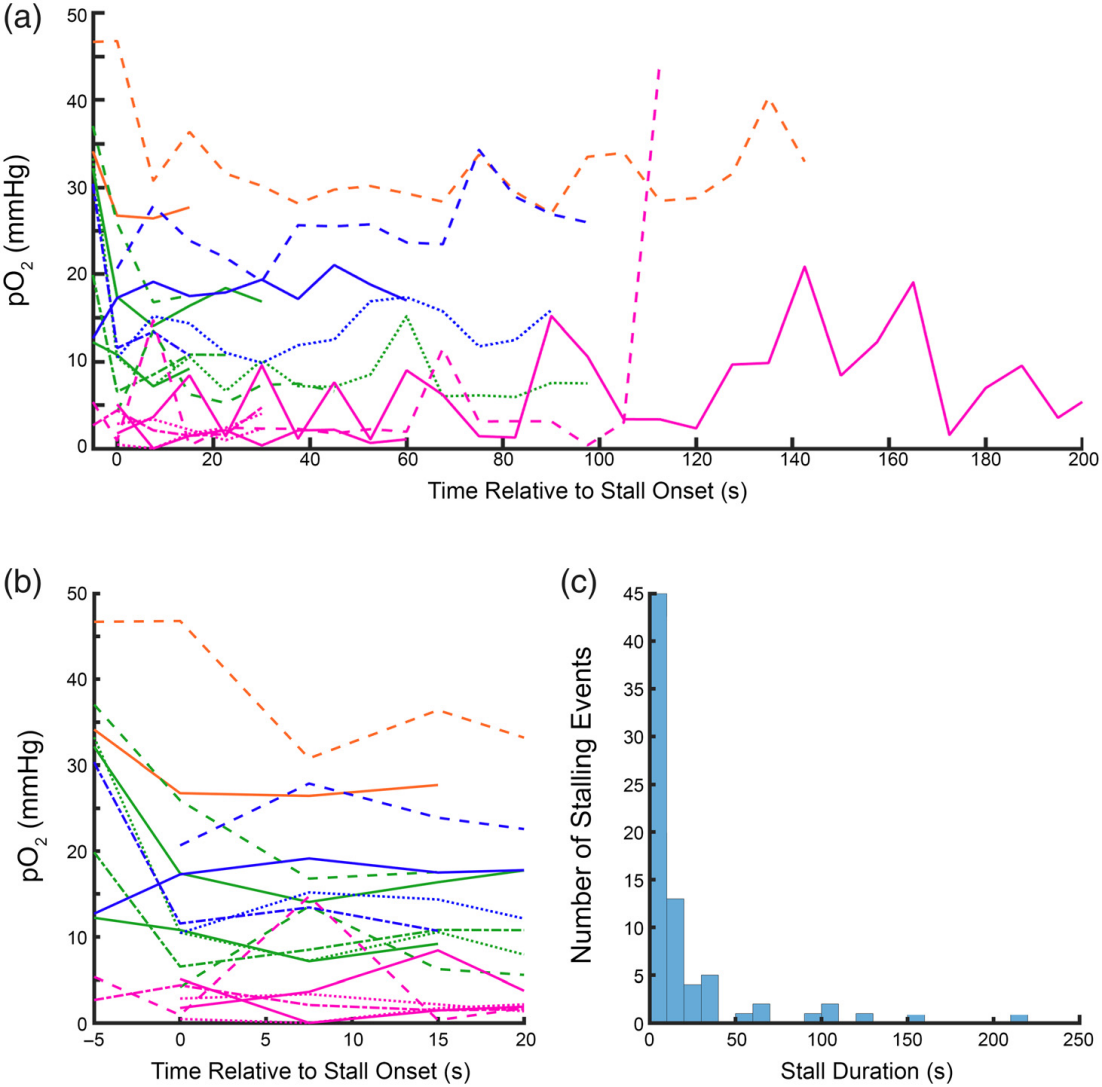
(a) Time traces of longer stalling events captured in this study. Each trace represents a single stalling event, with t=0 representing the first measurement where flux was stopped. Note, due to the long time between measurements (∼8  s), it is possible that the capillary began flowing and then re-stalled in between some time points. (b) Time traces from figure (a) are cropped to better show the immediate onset of the stalling events. (In both panels a and b, the same color indicates the same vessel. Different events are plotted in different styles to increase visibility). (c) Histogram of all stall durations measured, showing that the majority of events captured were brief in duration (typically one time point), consistent with measurements made by other modalities.^35^ Note some capillaries stall multiple times and contribute multiple events to the histogram.

Additional analyses on oxygen dynamics around capillary stalls. Figure 6 shows the relationship between stall cortical depth, showing no clear relationship between cortical depth and oxygen levels during a stall. Though, a complete loss of flux is not required to reach hypoxic oxygen levels, as demonstrated in the representative time trace shown in Fig. 7. We also show that for the small subset of longer stalls, stalls appear to reach a lower steady state that last the duration of the stall (Fig. 8). Finally, a table of all animals and measurements is provided, including the success rate of all PLIM measurements in providing “good” flux signal to enable capture of capillary stalling events.

**Table 1:** Summary of measurements taken across all mice and groups. “Good points” indicate measurements where both oxygen and RBC flux could be measured.

Group	Animals	Age (months)	Good points (stalls)	Total points measured
Anesthesia	3	4 to 6	114 (30)	798
Awake	7^a^	7 to 11	216 (32^b^)	867

a 3 additional animals were excluded due to poor data quality. 2 contributed 0 measurement points, and 1 contributed 3 total points (0 stalls).

b 2 animals did not have any stalls measured.

## Data Availability

Datasets used are available at https://doi.org/10.6084/m9.figshare.28541840.v1.
